# Angiogenesis in the Developing Spinal Cord: Blood Vessel Exclusion from Neural Progenitor Region Is Mediated by VEGF and Its Antagonists

**DOI:** 10.1371/journal.pone.0116119

**Published:** 2015-01-13

**Authors:** Teruaki Takahashi, Yuta Takase, Takashi Yoshino, Daisuke Saito, Ryosuke Tadokoro, Yoshiko Takahashi

**Affiliations:** 1 Department of Zoology, Graduate School of Science, Kyoto University, Kyoto, Japan; 2 Graduate School of Biological Sciences, Nara Institute of Science and Technology, Ikoma, Japan; 3 Frontier Research Institute for Interdisciplinary Sciences, Tohoku University, Sendai, Japan; 4 Core Research for Evolutional Science and Technology (CREST), Japan Science and Technology Agency (JST), Tokyo, Japan; University College London, UNITED KINGDOM

## Abstract

Blood vessels in the central nervous system supply a considerable amount of oxygen via intricate vascular networks. We studied how the initial vasculature of the spinal cord is formed in avian (chicken and quail) embryos. Vascular formation in the spinal cord starts by the ingression of intra-neural vascular plexus (INVP) from the peri-neural vascular plexus (PNVP) that envelops the neural tube. At the ventral region of the PNVP, the INVP grows dorsally in the neural tube, and we observed that these vessels followed the defined path at the interface between the medially positioned and undifferentiated neural progenitor zone and the laterally positioned differentiated zone. When the interface between these two zones was experimentally displaced, INVP faithfully followed a newly formed interface, suggesting that the growth path of the INVP is determined by surrounding neural cells. The progenitor zone expressed mRNA of *vascular endothelial growth factor-A* whereas its receptor *VEGFR2* and *FLT-1 (VEGFR1)*, a decoy for VEGF, were expressed in INVP. By manipulating the neural tube with either VEGF or the soluble form of FLT-1, we found that INVP grew in a VEGF-dependent manner, where VEGF signals appear to be fine-tuned by counteractions with anti-angiogenic activities including FLT-1 and possibly semaphorins. These results suggest that the stereotypic patterning of early INVP is achieved by interactions between these vessels and their surrounding neural cells, where VEGF and its antagonists play important roles.

## Introduction

Blood vessels form intricate networks that are widely distributed in the body. The vascular networks need to be properly patterned so that they efficiently supply oxygen, nutrients and physiologically active substances to tissues and organs. During blood vessel formation, vascular endothelial growth factor (VEGF) plays a pivotal role [1–8]. An increasing body of knowledge has revealed the mechanisms by which individual endothelial cells respond to VEGF [9–15]. However, it remains incompletely defined how the vascular patterning is regulated in growing tissues and organs during development.

The central nervous system (CNS; brain, retina, and spinal cord), where a considerable amount of oxygen needs to be supplied, serves as a good model to study vascular patterning [10,16,17]. During development of the brain and spinal cord, a vascular plexus forms and surrounds the CNS (peri-neural vascular plexus; PNVP), and this process is soon followed by an ingression of some PNVP cells into the CNS. Blood vessels that have ingressed into CNS are called the intra-neural vascular plexus (INVP) [4,10,18–23]. The INVP is stereotypically patterned [16,24,25], implying that environmental cues within the CNS play an important role in the patterning of this vasculature. In the forebrain and hindbrain of mice, the INVP ingresses through multiple entry points from the ventro-lateral surface of the brain, and grows inward along radial glia [26–28]. In the developing spinal cord, in contrast, the radially/laterally ingressing INVP is less obvious compared to that in the brain, and instead, the ventrally ingressing INVP has other characteristic behaviors: These blood vessels enter into the neural tube/spinal cord along the floor plate and grow in a dorsal direction; this type of INVP is hereafter called the ventro-dorsally growing INVP (vdINVP) to distinguish it from the latero-medially growing INVP (lmINVP). Whereas the initial entry of the lmINVP into the spinal cord has been shown to be influenced by VEGF [20], the regulation of vdINVP pattern is largely unexplored.

To understand the mechanisms underlying the patterning of the vdINVP, we visualized growing vasculature by fluorescent ink angiography [29], and we found that the growth of vdINVP was restricted to the defined path at the interface between the undifferentiated progenitor zone (facing the lumen) and differentiated zone (laterally positioned) in the neural tube. To identify the factors regulating and restricting these movements, we exploited advantages of chicken embryology wherein the neural tube can be electroporated with exogenous genes before INVP ingression. This enables specific manipulation of the environmental cues for the vdINVP. When the boundary between progenitor- and differentiated zones was experimentally displaced by mutant forms of Rho and Rac1, vdINVP faithfully followed the newly formed boundary. We also found that whereas *VEGF* mRNA was expressed in the progenitor zone, its receptor *VEGFR2* and the decoy receptor *FLT-1* (*VEGFR1)* [30–33] were co-localized in vdINVP. Neural-specific manipulations with pro-angiogenic VEGF or anti-angiogenic factors including a soluble form of FLT-1 and Semaphorin 3 members markedly affected the pattern of vdINVP migration. Thus, during development, the early patterning of the INVP appears to be controlled by surrounding neural cells, in particular, the progenitor zone, mediated by VEGF and its antagonists.

## Results

### Visualization of developing vascular plexus in the neural tube

**Figure 1:**
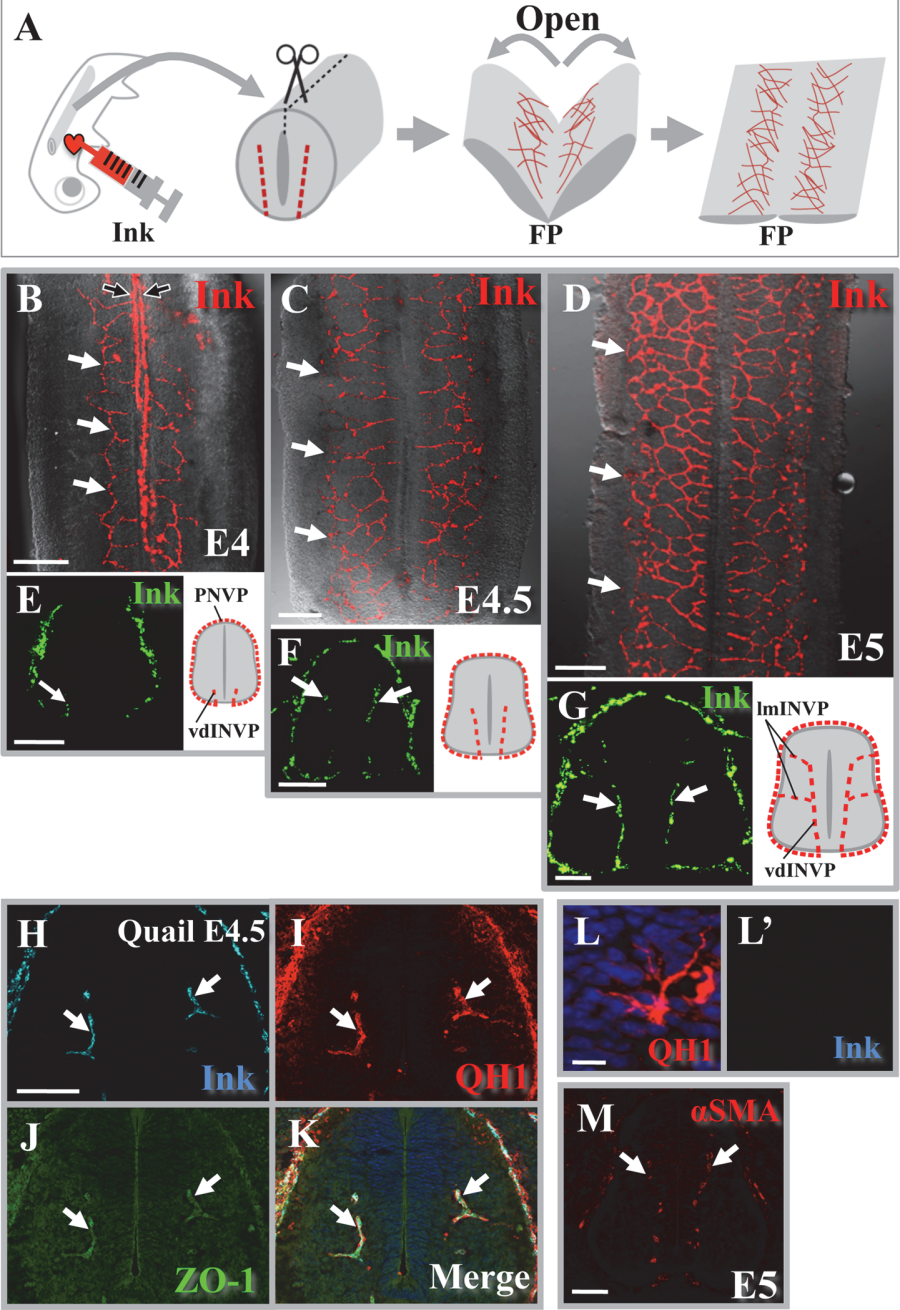
Growing vdINVP in chicken spinal cord visualized by fluorescent angiography. (A) A spinal cord was dissected from chicken embryos infused with fluorescent highlighter ink, followed by a flat-mounted preparation. (B-D) Flat-mounted preparation showing progressive growth of vdINVP (white arrows) after entering by the floor plate positioned in the middle. Stages; E4/HH22 in B, E4.5/HH24 in C, E5/HH26 in D. Longitudinal signal along the floor plate seen in B (black arrows) was due to incomplete removal of pial plexus. (E-G) Conventional transverse sections of the spinal cord prepared as shown in A. (H-K) Signals in a transverse section of quail spinal cord at E4.5 (corresponding to chicken E5/HH26) detected simultaneously by infused highlighter ink, QH1-staining, and ZO-1 staining. (L) Sporadically observed cells positive only for QH1 but not for infused highlighter ink in a quail spinal cord. (M) Chicken spinal cord of E5/HH26. Staining with anti-smooth muscle actin shows pericytes/mural cells (arrows) associated with growing vdINVP. FP: floor plate. Scale bars: 200 μm for (B-D), 100μm for (E-H, M), 10μm for (L).

To visualize the early patterning of INVP in the developing neural tube, we performed angiography using fluorescent ink (yellow highlighter ink; [29]) which highlights developing capillaries. From the trunk of each fluorescent ink-infused embryo, the neural tube was dissected out between the fore- and hindlimb buds. After incision along the dorsal midline (roof plate), the neural tube was laterally opened and subjected to a flat-mounted preparation (Fig. 1A). Thus, the lateral edges of the final specimen were originally the roof plate, whereas the original floor plate was in the center of the preparation. By embryonic day 4 (E4), fluorescent-labeled INVP started ingressing from the ventral side of the neural tube along both sides of the floor plate (Fig. 1B). This pattern is consistent with a previous report using Indian ink (non-fluorescent) and quail angioblast marker staining (QH-1) [20,22,24]. We also found that the ventrally ingressing blood vessels formed a plexus that progressively expanded in a dorsal direction as development proceeded (Fig. 1B–D; n = 8, 18, 20 for E4, E4.5 E5, respectively). Such progression was also observed in conventional histological transverse sections, although in these preparations the vascular plexus was often detected as discontinuous/punctate signals (Fig. 1E–G). By E5, the vdINVP connected to the lmINVP ingressing from the lateral aspect of the spinal cord (Fig. 1G), consistent with the previous study using QH-1 [20,22] (see also below). The dorsal-most portion of the neural tube was devoid of vascularization (Fig. 1B–G). Along the antero-posterior (AP) axis, entry points of vdINVP at the ventral edge of neural tube were distributed randomly (Fig. 1B–D; medially positioned longitudinal signals in Fig. 1B were due to the incomplete removal of the ventral pial plexus that lies outside this region, and they were not in register with somite segmentation pattern, as previously reported using black Indian ink [24]).

We recently reported that infused highlighter fluorescent ink visualizes developing vasculature in a whole embryo, and also that the fluorescent signal is retained after fixation and section preparation [29]. We therefore carefully compared highlighter-labeled vdINVP with immuno-histochemically detected signals for QH-1 (a marker for angioblasts, macrophages and endothelial cells in quail embryos [20,22,34–37]), ZO-1 (a marker for the tight junctions in the endothelium of blood vessel lumens), and smooth muscle actin (SMA) (a marker for pericytes/mural cells [36–38]) in transverse histological sections. As shown in Fig. 1H–K, in a quail neural tube at E4.5 (equivalent of chicken E5), the patterns of QH-1 and fluorescent ink signals largely overlapped (Fig. 1H, I; n = 8). QH1-positive and fluorescent-negative cells were also sporadically observed (Fig. 1L). It is likely that these cells were immature angioblasts and/or tip cells. Fluorescent ink signal coincided with ZO-1 staining (Fig. 1J, K), and was accompanied with SMA-positive pericytes/mural cells (Fig. 1M; n = 15). Together, angiography with highlighter successfully labeled the forming INVP with fluorescent signals.

In addition, we prepared a thick transverse section (70 μm) of highlighter ink-infused neural tube, and subjected it to confocal microscopy to reconstruct a 3D image. As shown in S1 Movie, the vdINVP expanded dorsally as a plexus structure. Such structures are reminiscent of growing vasculature in neonatal retina [13,39–41]. In the following experiments, the developing INVP was visualized by highlighter angiography.

### vdINVP grew along a defined path excluded from the neural progenitor zone

**Figure 2:**
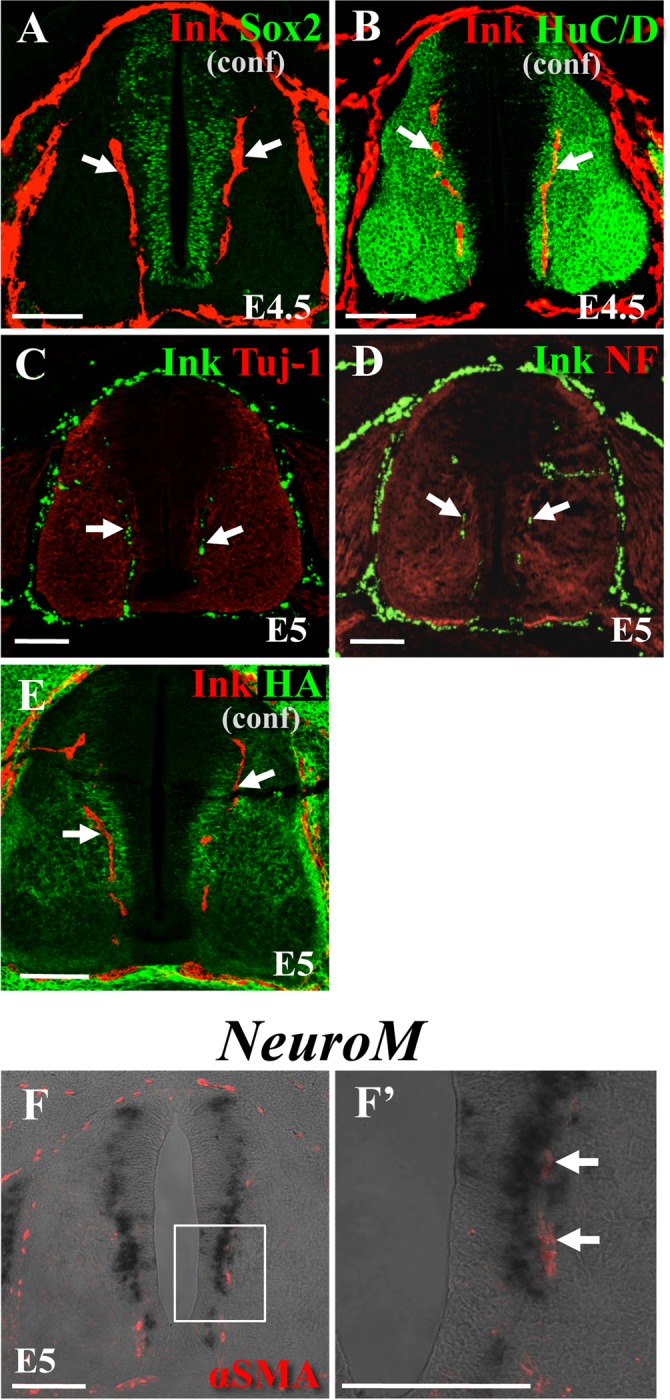
Comparison between the positions of vdINVP and different zones of neural cells in chicken spinal cord. (A) A transverse section of highlighter ink-infused spinal cord was co-stained with the progenitor marker Sox2. (B-E) Sections were stained for markers for differentiated zone, HuC/D, Tuj-1, NF, and HA. For A, B, and E, and 100 μm thick section was processed with confocal microscopy. Sections shown in C and D were 10 μm thick photographed by the conventional fluorescent microscope (Apotome-processed). (F) Growing INVP (arrows) stained with αSMA was located by the *NeuroM*-positive zone revealed by section in situ hybridization. F’ is a magnified view of the square in F. conf: a confocal image obtained by Z-stack for 70 μm out of 100 μm histological section. Arrows: growing vdINVP. (A, B) E4.5/HH24. (C-F) E5/HH26. Scale bars: 100 μm.

In early developing spinal cord at E4∼E5, neural cells of different stages of differentiation are positioned in distinct zones: undifferentiated/progenitor cells are in the medial-most zone facing the lumen, whereas the more differentiated neurons are positioned laterally near the pial surface [42–45]. We noticed that the growing path of the vdINVP was correlated with the border delineating the neural progenitor zone. To clarify the spatial relationships between the INVP and the progressive neural differentiation states in the spinal cord, we compared the position of vdINVP with the undifferentiated/progenitor marker Sox2, and with markers for the more differentiated zone, including Tuj-1, HuC/D, neurofilament (NF), and hyaluronic acid (HA) [46] (Fig. 2A–E; n = 8, 3, 10, 6 and 8 for A, B, C, D, E, respectively). The INVP was excluded from the Sox2-positive neural progenitor zone (Fig. 2A), and localized in the medial-most region of differentiated zone stained for Tuj-1, HuC/D, NF, and HA (Fig. 2B–E). Furthermore, the vdINVP was juxtaposed to the lateral border of NeuroM-positive area, known to be a marker for the intermediate zone positioned in between the progenitor and differentiated zones [44,45] (Fig. 2F; n = 5). These observations raised the possibility that the growing path of vdINVP would be determined by the surrounding environment, most likely by the differentiation states of neural cells in the neural tube.

### Disturbance of neural progenitor zone altered the pattern of INVP

**Figure 3:**
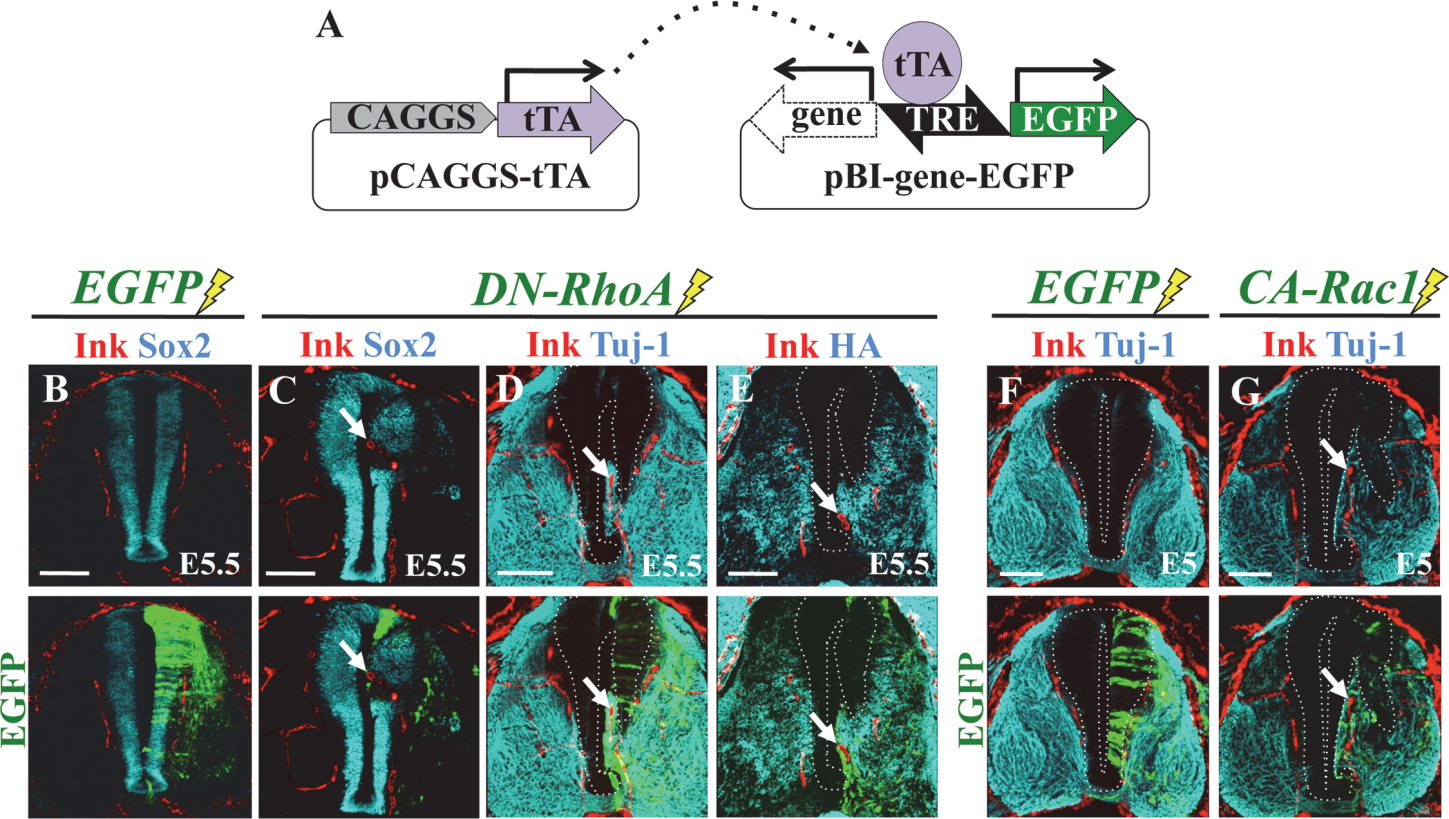
Disturbance of the neural progenitor zone altered the pattern of vdINVP. (A) Schematic illustration of pBI-based expression vectors. pBI-vector and pCAGGS-tTA were used without Dox, the combination that retained expression of electroporated DNAs. (B) Control electroporation with EGFP. (C-E) DN-RhoA was electroporated unilaterally into the neural tube. Thick transverse sections were prepared from highlighter ink-infused embryos and co-stained for markers indicated, followed by confocal microscopy. Top and bottom panels are identical views. (F, G) Similar experiments using CA-Rac1. Arrows: vdINVP misdirected into a lumen-facing region. (B-E) E5.5/HH27. (F, G) E5/HH26 Scale bars: 100 μm.

To understand the mechanism for the positioning of the vdINVP, we experimentally perturbed the neural progenitor zone and examined how this manipulation would affect the placement of the INVP. It has been known that the Rho family GTPases and their related proteins are involved in establishing distinct zones of neural differentiation states in developing CNS [47–49]. We therefore electroporated dominantly negative RhoA (DN-RhoA) unilaterally into an E2 neural tube. To trace electroporated cells, the pBI-based expression vector [50] was used wherein tetracycline-responsive element (TRE) bidirectionally drives EGFP and DN-RhoA. Thus, pBI-DNRhoA-EGFP was co-electroporated along with pCAGGS-tTA, which encodes a transcriptional activator for TRE (Fig. 3A). As expected, electroporated cells underwent precocious differentiation, and the lumen-facing region (normally Sox2-positive) was replaced by Tuj-1-positive cells endowed with more differentiated characters (at E5, Fig. 3B–E; n = 8, 6, 24, 10 for B, C, D, E, respectively). Notably, blood vessels invaded the newly formed Tuj-1-positive area. Electroporation with constitutively active Rac1 (CA-Rac1) resulted in a similar phenotype (Fig. 3F, G; n = 6 for F; n = 10 for G). These observations suggested an intimate correlation between the formation of the vdINVP and neural differentiation states.

### Expression patterns of *VEGF, VEGFR2*, and *FLT-1/VEGFR1* in growing vdINVP

**Figure 4:**
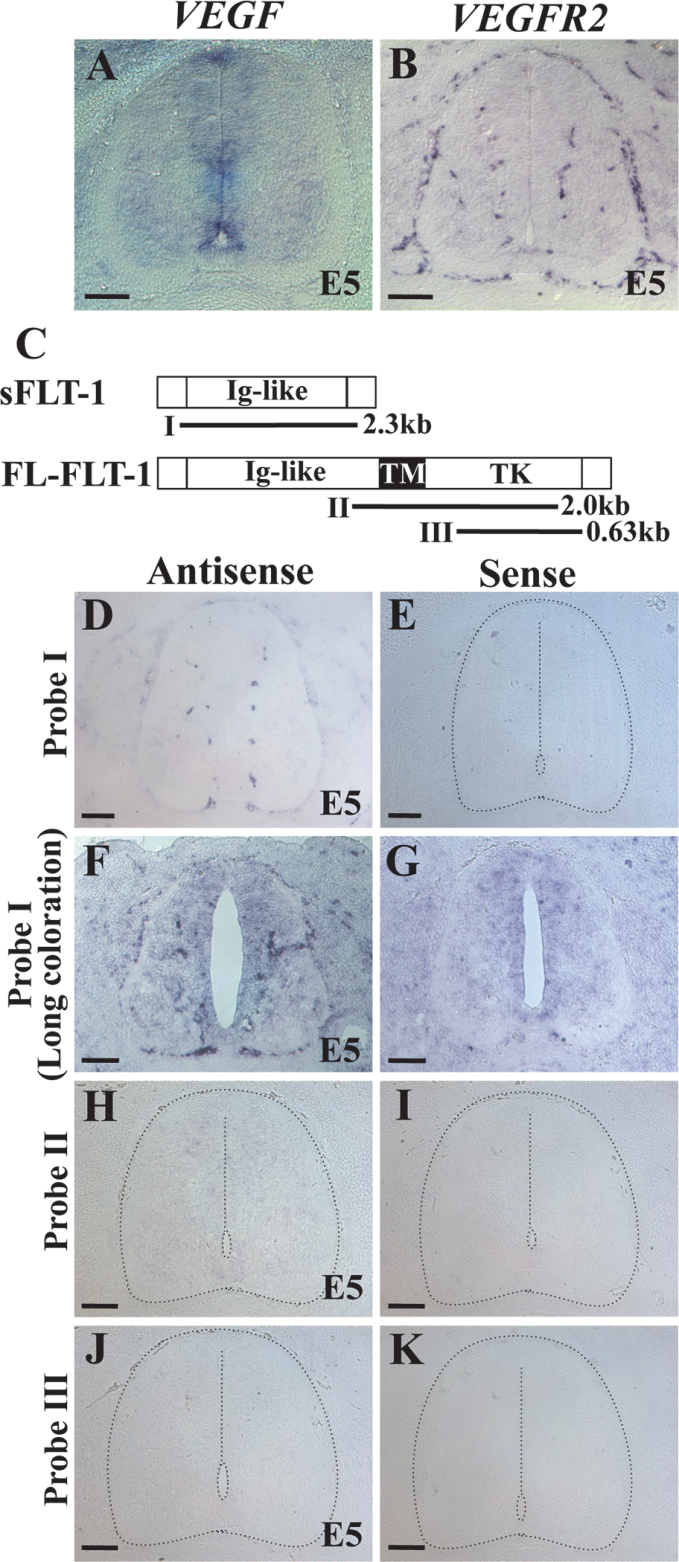
mRNA expression patterns of *VEGF, VEGFR2*, and *FLT-1/VEGFR1* in growing vdINVP analyzed by section in situ hybridization. (A) *VEGF* mRNA. (B) *VEGFR2* mRNA. (C) *FLT-1/VEGFR1* mRNA. Probe I was prepared from *soluble FLT-1*, and probes II and -III detecting cytoplasmic region were from full length (fl) *FLT-1* cDNA. (D-K) Except F and G, which were of long coloration time to detect digoxigenin (5 days), coloration was terminated after 3 days. Probe I antisense yielded signals in INVP (D). Faint signals in the progenitor zone in F appears to be a noise since the sense probe also gave similar staining (G). Probes II and -III failed to give signals (H-K). Specimens are transverse sections of chicken spinal cord at E5/HH26.

To further understand the molecular mechanisms by which the growing path of the vdINVP is determined, we paid attention to VEGF, the most prominent angiogenic factor, and to its receptors. It was known [19,51–53] and was also confirmed in the current study by in situ hybridization that mRNAs for *VEGF* and its receptor *VEGFR2* are expressed in the neural progenitors and in the vdINVP, respectively, in the E5 spinal cord (Fig. 4A, n = 8; 4B, n = 4). We further examined expression patterns of *FLT-1/VEGFR1*, predicted to encode a decoy receptor that interferes with VEGF-VEGFR2 signaling [30–33]. Whereas a probe for the extra cellular domain of *FLT-1* (Probe I) yielded signals in endothelial cells of the vdINVP, the probes covering the cytoplasmic region (Probe II and III) gave no signals (Fig. 4C–K, n = 8 for each). Although the progenitor zone was faintly stained by antisense probe I after prolonged time, such signals were also obtained by the sense probe (Fig. 4F, G). Thus, we conclude that the INVP is the sole tissue expressing *FLT-1* in the embryonic spinal cord at E5. It is known that *FLT-1* encodes two alternative splicing variants, one encoding the soluble form (which is extracellular) and the other encoding the full-length form (which is a transmembrane protein) [20,54–56]. Thus, it is conceivable that the *FLT-1* expressed in the INVP encodes the soluble form and may act as a decoy for VEGF signaling through VEGFR2.

### The vdINVP grows in a VEGF-dependent manner

**Figure 5:**
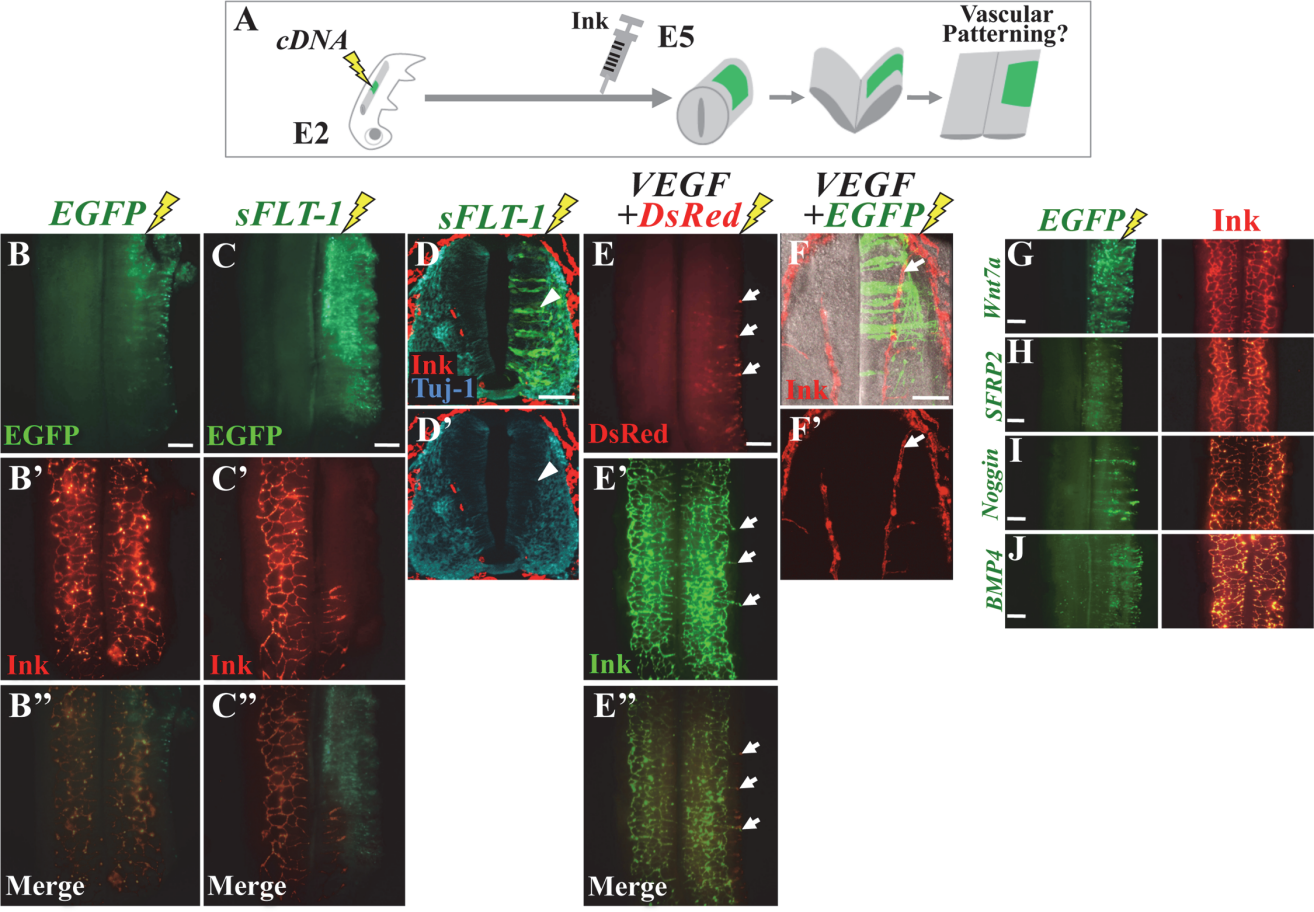
vdINVP grew in a VEGF-dependent manner. (A) Experimental design to examine effects by sFLT-1 or VEGF. cDNAs were electroporated unilaterally into the neural tube at E2 (HH14), and manipulated embryos were infused with highlighter ink at E5/HH26, flowed by flat-mounted preparation. (B) Control electroporation with EGFP. (C) *sFLT-1* was co-electroporated with EGFP. vdINVP was excluded from the sFLT-1/EGFP positive area. (D) The inhibitory action by sFLT-1 was also observed in conventional transverse sections (arrowhead). *sFLT-1*-overexpression did not cause gross effect at least on the progenitor zone. (E) *VEGF* co-electroporated with *DsRed* caused ectopic vascularization extending to VEGF-electroporated sites (arrows). Such hypervascularization (arrow) was also confirmed in a transverse view processed for 70 μm thick (Z-stack) by confocal microscopy, where growing plexus could be seen as continuous tissue (similar to Fig. 2 A, B, E) (F). (G-J) Similar manipulations with four different cDNAs as indicated (flat-mounted preparation). pBI-Wnt7a-EGFP/pCAGGS-tTA, pBI-SFRP2-EGFP/pCAGGS-tTA, pCMS-Noggin-EGFP, and pCMS-BMP4-EGFP, were tested. No gross effects were appreciated on the vdINVP patterning. Scale bars: 100 μm for (D, F), 200 μm for (B, C, E, G–J).

To determine whether and how the growth of the vdINVP is regulated by VEGF, we overexpressed into an E2 neural tube an expression vector that carried a cDNA encoding a soluble form of FLT-1 (sFLT-1: the extracellular domain of FLT-1) that would interfere with VEGF-VEGFR2 signaling [20,54–56] (Fig. 5A). As in Fig. 3, pBI-sFLT-1-EGFP was co-electroporated with pCAGGS-tTA without Dox. We found that the sFLT-1-electroporated region was devoid of vascularization both in flat-mounted specimens (Fig. 5B, control, n = 24; Fig. 5C, sFLT-1, n = 13) and in conventional transverse sections (Fig. 5D, n = 12). No gross effects on neural differentiation zones were observed by sFLT-1 overexpression (Fig. 5D), consistent with the previous report [20].

Conversely, when we co-electroporated a cDNA encoding mouse *VEGF-164* along with the *DsRed* or *EGFP* cDNA into a dorsal part of the neural tube that would normally remain avascular (Fig. 1D), blood vessels extended to VEGF-overexpressing regions. This was observed both in flat-mounted specimens and in transverse sections of the E5 spinal cord (Fig. 5E–F; n = 21). Thus, the vdINVP was positively regulated by VEGF.

We also asked whether vdINVP would be affected by other extracellularly acting factors implicated in angiogenesis. Wnt7/β̃catenin signals have been reported to be required for blood vessel formation and/or their maturation in the central nervous system [23]. Electroporation with Wnt7a or SFRP2 (secreted Frizzled-related proteins: Wnt inhibitor) yielded no significant effects on vdINVP ingression or growth (Fig. 5G, n = 5; 5H, n = 4). BMPs were also shown to be an angiogenic factor for the development of axial veins in zebrafish [57], but neither BMP4 nor its inhibitor, Noggin, affected vdINVP patterning when electroporated into a neural tube (Fig. 5I, n = 4; 5J, n = 10).

We thus conclude that VEGF plays a role as a major pro-angiogenic factor in vdINVP patterning. Since endothelial cells of the vdINVP co-express VEGFR2 and FLT-1, it is conceivable that the VEGF2-mediated pro-angiogenic action is fine-tuned by anti-angiogenic FLT-1 signals (also see Discussion). Such counteractions were previously reported for regulating the entry points of laterally ingressing blood vessels from PNVP [20].

### Overproduced INVP in the progenitor zone caused hemorrhage into the lumen

**Figure 6:**
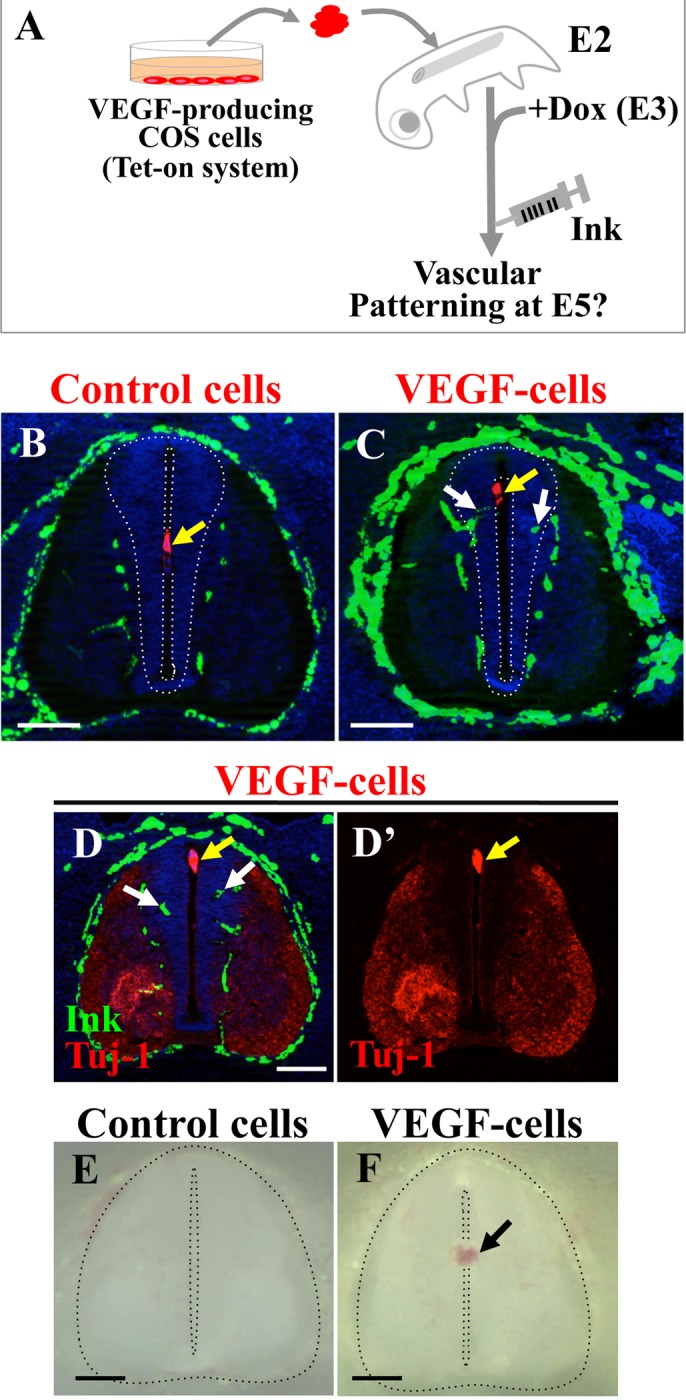
Implantation with VEGF-producing cells into the lumen of neural tube resulted in hemorrhage. (A) Experimental design showing that an aggregate of VEGF-producing COS cells was implanted into an E2 (HH14) embryo, which was harvested at E5/HH26. Production of VEGF was controlled by the tet-on system so that the expression started at E3 (HH18) by Dox administration. (B, C) Implanted VEGF-COS cells (yellow arrow in C) caused invasion of vdINVP into the progenitor zone (white arrows in C), whereas control COS cells (yellow arrow in B) yielded no effects. (D) Tuj-1 staining showed no gross effects on neural differentiation by VEGF-COS cells (yellow arrow in D). White arrows show ink-infused vdINVP in the progenitor zone. (E, F) Implanted VEGF-COS cells caused hemorrhage (arrow in F), whereas control COS cells yielded no effects (E). Scale bars: 100 μm.

To get insight into the physiological significance of the avascularization in the progenitor zone, we attempted to experimentally vascularize this region. For this purpose, we implanted an aggregate of VEGF-transfected COS cells (stable cell line; see Materials and Methods) into the lumen of the neural tube at E2 (Fig. 6A). Production of VEGF was temporally controlled by the tet-on system so that the expression started at E3 by Dox administration. As expected, this manipulation caused an invasion of blood vessels into the progenitor zone without gross influence on the neural zone alignments assessed at E5 (Fig. 6B–D, n = 12, 10, 8 for B, C, D, respectively). Notably, hemorrhage occurred in the lumen of the spinal cord (Fig. 6E, F, n = 4 for each). It is conceivable that during development the progenitor zone may act as a barrier to protect the cerebrospinal fluid from influx of systemic blood. Alternatively, the progenitor zone may be incapable of providing the environment to form the blood-brain barrier (BBB) with the INVP.

### The vdINVP responded to repellant action of Sema3E but not of Sema-3A, -3B, and -3C

To see whether additional inhibitory factors might be preventing the vascular invasion into the VEGF-positive progenitor zone, we examined the effects of Semaphorin 3 (Sema3) family members that have been implicated in regulating angiogenesis. Among Sema3 members, Sema3E has been reported to inhibit angiogenesis in mice [58–64], but little information is available for chicken vascular formation.

**Figure 7:**
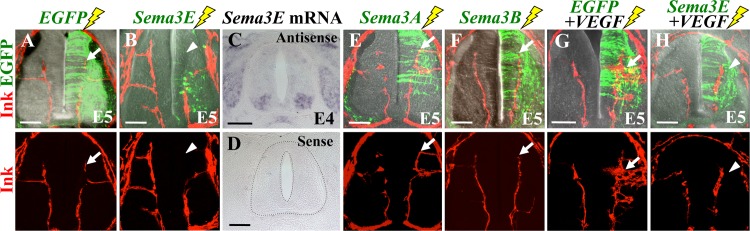
Sema3E, but not Sema3A or -3B, exerted inhibitory actions on growing vdINVP. Transverse sections were prepared from highlighter ink-infused embryos. (A) Control electroporation with EGFP. Arrow shows vdINVP. (B) Electroporation with *Sema3E* inhibited INVP (arrowhead). (C, D) In situ hybridization for *Sema3E* mRNA in spinal cord at E4 (HH22). Motor columns were positive. (E, F) Electroporation with *Sema3A* or *Sema3B* yielded no effects on vdINVP formation (arrows). (G, H) *VEGF+EGFP*, or *VEGF+Sema3E* were electroporated into the neural tube. *Sema3E*-overexpression neutralized VEGF-induced hypervascularization of vdINVP (arrowhead in H). Except C and D, specimens were of E5/HH26 subjected to confocal microscopy for Z-stack of 70 μm thick. Top and bottom panels are identical views. Scale bars: 100 μm.

By the in ovo electroporation (see Fig. 5), we overexpressed each of *Sema3A* (chicken), *Sema3B* (human), *Sema3C* (chicken), and *Sema3E* (chicken) into the chicken neural tube along with *EGFP* at E2. Each of these *Sema* genes was carried by the pBI-EGFP vector. We found that Sema3E markedly inhibited the invasion of the vdINVP into the electroporated side of E5 spinal cord (Fig. 7A, B; n = 9). In contrast, none of other Sema3 members exerted detectable effects (Sema3A, Fig. 7E, n = 10; Sema3B, Fig. 7F, n = 7; Sema3C, not shown, n = 6) [65]. Previous studies reported that among Sema3 family members, Sema3E is the sole member that directly binds to Plexin receptor to transduce repulsive signals whereas other Sema3 members bind to the co-receptor Neuropilin, which subsequently associates with Plexin. PlexinD1 is known to be expressed in the INVP [66]. However, we failed to detect expression of *Sema 3E* mRNA in the progenitor zone although the signal was successfully detected in the motor columns (Fig. 7C, D) as previously reported for mice [67]. Thus, although it is unlikely that Sema3E acts as an endogenous repellent, it is conceivable that the INVP receives inhibitory Sema signals other than those we have tested (see Discussion).

To further see whether there were complex interactions between VEGF-mediated angiogenesis and Sema/Plexin-mediated anti-angiogenesis, we compared *VEGF*-electroporated- and *VEGF*+*Sema3E*-electroporated specimens. In both experiments, *EGFP* cDNA was co-electroporated to visualize the electroporated region. Whereas VEGF-overexpression yielded hyper-vascularization as shown above (Fig. 7G), co-electroporated Sema3E neutralized this hyper-vascularization (Fig. 7H). Thus, it is conceivable that during normal development, VEGF-mediated angiogenesis could be fine-tuned by counteracting activities of both FLT-1 and Sema/Plexin, and such combinatorial regulations might determine the stereotypic path of the growing vdINVP.

## Discussion

### The growing path of vdINVP is determined by surrounding neural cells in the developing spinal cord

We have demonstrated that the stereotypic patterning of the vdINVP is achieved by environmental cues in the developing spinal cord. The vdINVP expands dorsally as a planar structure along the defined path confined to the narrow intermediate zone (NeuroM^+^) that is positioned in between the progenitor zone (Sox2^+^) and differentiated zone (TuJ1^+^, NF^+^, HuC/D^+^) (Fig. 2). When the interface between these zones was experimentally displaced, the vdINVP faithfully followed the newly formed boundary (Fig. 3). Notably, the vdINVP never invades the progenitor zone in normal- and neural zone-manipulated embryos. However, *VEGF* mRNA, shown to be required for the INVP growth in this study, was confined to the progenitor zone (Fig. 4). We propose the possibility that the VEGF signal is fine-tuned by interactions between pro-angiogenic signaling via the VEGFR2 receptor and the anti-angiogenic FLT-1, both of which are expressed in the INVP. Thus, the signal level of VEGF would be sufficient for the growth/patterning but not for the invasion of the vdINVP into this zone. Indeed, when the progenitor zone was overwhelmed by VEGF using VEGF-producing COS cells, the vdINVP was able to invade this zone (Fig. 6). Since FLT-1 expressed in the INVP appears to be a splicing variant containing only the extracellular region (Fig. 4C), probably a soluble form [20,54–56], this isoform may act as a decoy that traps extra amount of VEGF. How the VEGF protein is distributed in the spinal cord awaits further investigation.

Although the avascular progenitor zone was previously shown by QH-1 staining [20,21], our current study has provided the first evidence of precise comparisons between blood vessels and distinct neural zones in both normal- and zone-manipulated embryos. Changes in the pattern of the INVP when the border of progenitor zone was displaced (experiments with DN-RhoA/CA-Rac1) could be attributed to changes in cell adhesion of neural cells. However, since the invasion of the INVP was also seen in VEGF-implanted neural tube (Fig. 6), in which neural progenitor cells were not affected, it is unlikely that loosened cells were the primary cause of the INVP invasion. Rather, it is likely that changes in cell adhesion affected cell differentiation states, which in turn lead to the misrouting of the INVP.

### Possible roles of Sema family in the vdINVP patterning

We have presented that the vdINVP is inhibited by Sema3E, but not by Sema3A, Sema3B or Sema3C. Sema3E has been known to act as an anti-angiogenic molecule critical for proper formation of vasculature in mice [58–64]. Unlike other Sema3 family members that signal through Plexin via Neuropilin, Sema3E binds directly to PlexinD1. Since PlexinD1 is shown to be expressed in the INVP [66], it is conceivable that Sema-Plexin signal plays an important role in the avascularization of the progenitor zone and/or defining the growing path of the vdINVP. Thus, the vdINVP appears to be regulated by complex counteracting mechanisms to which Sema-Plexin and the decoy FLT-1 contribute. Sema3E may not be an endogenous inhibitor since we failed to detect *Sema3E* mRNA in the progenitor zone (Fig. 7C, D). Of note, Sema6A, which binds directly to Plexin like Sema3E, was reported to be expressed in the progenitor zone in mice [68]. Further studies are needed to determine combinatory interactions between VEGF and anti-angiogenic factors.

### Avascularization of the progenitor zone of CNS

In this study, we have highlighted the avascularization of the progenitor zone in the developing spinal cord. We have also demonstrated that ectopic vascularization caused by a high level of VEGF resulted in hemorrhage into the lumen. This raises the possibility that the progenitor zone needs to be avascular to protect cerebrospinal fluid against influx of systemic blood. Alternatively, the progenitor zone might not harbor environment that allows formation of BBB to prevent hemorrhage.

The vascular-free space is also seen in the hindbrain, where the subventricular zone (SVZ) is devoid of blood vessels [26]. In the hindbrain, the vdINVP is not prominent, and most INVP is ingressed laterally and grows inward until reaching the border of the SVZ, where its tip cells subsequently turn 90 degrees to connect to the neighboring INVP [9]. Yet, similar mechanisms might be shared in the brain and spinal cord so that the lumen-facing zone acts as a barrier to prevent hemorrhage in the lumen. And to achieve this, balance between VEGF and multiple anti-angiogenic factors might be important. In this context, the findings provided in this study could help therapeutic treatments of diseases related to cerebro-spinal fluid dysfunction and also brain infarction.

## Materials and Methods

### Ethical approval

Ethical approval for this study has been acquired (No. H2620, Kyoto University).

### Chicken and quail embryos

Fertilized chicken and quail eggs were commercially obtained from the poultry farm Shiroyama Farm (Kanagawa, Japan) and Motoki Corporation (Saitama, Japan), respectively. Embryos were staged according to Hamburger Hamilton (HH stage) [69]. Embryonic day 2 (E2) corresponds to HH14, and E3, E4, E4.5, E5, and E5.5 to HH18, HH22, HH24, HH26, and HH27, respectively.

### Angiography using highlighter fluorescent ink

In vivo visualization of blood vessels was performed as previously described [29]. Chicken embryos were infused with 1–3 μl highlighter ink (PILOT spotliter; 100 x dilution in phosphate buffered saline (PBS) through vitelline artery using a micropipette with the tip of 1 μm diameter prepared from a glass capillary (Narishige, GD-1) using a vertical micropipette puller (Narishige, PC-10).

### Flat-mounted preparation of the spinal cord

A flat-mounted preparation of the spinal cord was carried out essentially as previously reported [70,71] with slight modifications. Spinal cord was dissected from an ink-infused embryo, and PNVP was carefully removed using tweezers and a micro feather blade (FEATHER). The PNVP-free spinal cord was laterally opened after incision along the roof plate, and transferred to a 35 mm glass bottom dish (MATSUNAMI) with the luminal side facing down for confocal microscopy.

### Immunohistochemistry

Embryos were fixed overnight in PBS containing 4% paraformaldehyde (PFA) at 4°C, followed by preparation of cryostat sections of 10 μm or 100 μm thick.

#### For 10 μm thick sections

After washing in PBS and blocking with 2% skim milk/PBS for 1 h at room temperature (RT), the sections were incubated overnight at 4°C with diluted antibodies in 2% skim milk/PBS; 1:1 of culture supernatant of QH-1 mouse monoclonal antibody (DSHB), 1:50 of anti-ZO-1 rabbit polyclonal antibody (Zymed) [72], 1:400 of anti-smooth muscle actin (αSMA) mouse monoclonal antibody (1A4; Sigma), 1:300 of anti-β-tublinIII (Tuj-1) mouse monoclonal antibody (R&D systems), 1:200 of anti-Neurofilament rabbit polyclonal antibody (Millipore). After washing three times in PBS, specimens were reacted with Alexa 568 goat anti-mouse IgG, Alexa 488 goat anti-rabbit IgG or Alexa 647 goat anti-mouse IgG (Molecular Probes) with 1:500 dilution for 1 h at RT. The reaction was terminated by washing three times in PBS, and the sections were sealed by FluorSave reagent (Calbiochem).

#### For 100 μm thick sections

Sections were treated in suspension in the non-coating 24-well plate (IWAKI). After washing in PBS containing 0.5% Triton X-100 and blocking with 2% skim milk/PBST (PBS containing 0.1% Tween 20) for 2 h at RT, the sections were incubated overnight at 4°C with diluted antibodies in 2% skim milk/PBST; 1:50 of anti-Sox2 goat polyclonal antibody (R&D systems), 1:100 of anti-HuC/D mouse monoclonal antibody (Molecular Probes), 1:300 of anti-β-tublin III (Tuj-1) mouse monoclonal antibody (R&D systems). After washing three times in PBST, the specimens were reacted with Alexa 488 donkey anti-goat IgG, Alexa 488 goat anti-mouse IgG or Alexa 405 donkey anti-goat IgG (Molecular Probes) with 1:500 dilution for 1 h at RT. The reaction was terminated by washing three times in PBST, and the sections were sealed by FluorSave reagent (Calbiochem).

### Histochemical detection of Hyaluronan

Hyaluronan (HA) was detected by a biotinylated HA-binding probe (HABP, Hokudo). The histochemical reaction in the spinal tissues was carried out as previously described [46]. The reaction was visualized with Streptavidin-Alexa 555 or Streptavidin-Alexa 647.

### Expression vectors

pBI-EGFP (Clontech), pCAGGS-tTA, pBI-DN-Rac1, pBI-DN-RhoA were used as previously described [50]. Chicken cDNA of sFLT-1 was gifted by Dr. Shibuya [56], and subcloned into pBI-EGFP. pCAGGS-DsRed express, pCAGGS-VEGF164 (mouse) and pCAGGS-EGFP were used as previously described [37]. Chicken Wnt7a cDNA (1,050 bp, GenBank NM_204292) was isolated by using the following primers, attACGCGTatgaacaggaaaacaaggcg (Fw-MluI-Wnt7a) and tctGATATCacttacaggtatatactt (Rv-EcoRV-Wnt7a), and subcloned into pBI-EGFP. pBI-SFRP2-EGFP (chicken: 879 bp, GenBank NM_204773) was constructed by Dr. Shimokita. pCMS-BMP4-EGFP and pCMS-Noggin-EGFP were as previously described [73]. Human cDNA of Sema3B (Sino Biological) was subcloned into pBI-EGFP.

The ORFs of chicken Semaphorin 3 family were isolated by PCR using following primer sequences. Isolated cDNAs of Semaphorin 3 family were separately subcloned into pBI-EGFP.

Fw-MluI-Sema3a (GenBank NM_204977)

atACGCGTtacagtggctgctgcagcatg

Rv-EcoRV-Sema3a

atGATATCtcagacactccgtggtgcc

Fw-MluI-Sema3c (GenBank NM_204243)

atACGCGTctgaagagatggcagttctt

Rv-EcoRV-Sema3c

atGATATCttaagaagcaggtaactgatttc

Fw-NheI-Sema3e (GenBank NM_204242)

atGCTAGCgccgccatgttgggcaggatggca

Rv-NheI-Sema3e

atGCTAGCtcaagagtcagctatgttcctc

### In ovo electroporation

In ovo electroporation was carried out according to the method previously reported [73–76] with slight modifications; Anode and cathode were prepared with a platinum wire (diameter of 0.5 mm) and a tungsten needle (diameter of 0.5 mm), respectively. DNA plasmids were diluted in EB buffer (QIAGEN) containing 4% fast green FCF (Nacalai) at a final concentration of 6 μg/μL. The DNA solution was injected into the neural tube cavity of E2 embryos. Subsequently, electric pulses of 10–15 V, 25 ms were applied five times with 975-ms intervals.

### In situ hybridization

A 993 bp fragment of chicken NeuroM cDNA (GenBank NM_205076) was isolated by PCR using following primer sequences; Fw-atACGCGTgccgccatgacgaagacgtacaccaa, and Rv-atGCTAGCctactcgttgaagatggcgt. An amplified fragment was digested by MluI-NheI and subcloned into pCMS-EGFP, which had been treated with MluI- NheI [73]. cDNA fragments for in situ probes of chicken VEGF (0.6 kb, GenBank NM_205042) and quail VEGFR2 (Quek1; 0.5 kb, GenBank X83288) were isolated by Dr. Tonegawa. Chicken cDNA of sFLT-1 (2.3 kb) was gifted by Dr. Shibuya [56], and subcloned into pCMS-EGFP. For examining the expression of cytoplasmic region, two kinds of in situ probes were prepared; a 2.0 kb fragment containing the transmembrane region (probe II), and 0.63 kb corresponding to a 3’ region (probe III) (Fig. 4C). cDNAs of respective regions were PCR-amplified based on *FLT-1* sequences (GenBank NM_204252) using primers as follows; for probe II (2.0 kb), Fw-atACGCGTatctggtattgtcctgttca, Rv-atGCTAGCgcttggtagtcgttaaatac; for probe III (0.63 kb), Fw-atACGCGTatctgagaacaatgtagtga, Rv-atGCTAGCgcttggtagtcgttaaatac. Amplified fragments were digested by MluI-NheI and subcloned into pCMS-EGFP. Preparation of digoxigenin-labeled probes and in situ hybridization were performed as previously described [77]. Coloration to detect digoxigenin was terminated after 3 to 5 days.

### VEGF -producing COS cells

COS7 cells derived from the kidney of the African Green Monkey (ATCC) were maintained at 37°C with Dulbecco’s modified Eagle’s medium (DMEM) containing 1.5 g/L sodium bicarbonate, 10% fetal bovine serum (FBS), 50 IU/ml penicillin (Banyu Seiyaku), and 50 mg/ml streptomycin (Meiji). pT2K-BI-TRE-Gap43-tdTomato-VEGF164 was co-transfected with pCAGGS-T2TP and pT2K-rtTA2^s^M2-IRES-NeoR [50,78] into COS7 cells using Lipofectamine 2000 (Invitrogen) according to the manufacture’s instruction. For control experiments, pT2K-BI-TRE-Gap43-tdTomato was used instead of pT2K-BI-TRE-Gap43-tdTomato-VEGF164. After transfection, cells were cultured in G418-containing medium for 17 days, and among G418-resistant colonies Gap43-tdTomato-positive clones were selected under fluorescent microscope. A solution of Doxycycline (an analog of tetracycline; Dox) (0.5 ml of 0.1 μg/μl) was injected into an egg yolk one day after implantation of VEGF-producing COS cells into the lumen of neural tube of E2 embryo.

### Microscopy and image processing

Images for flat-mounted specimens were obtained using LSM5 Pascal confocal laser scanning microscope (Carl Zeiss), stereomicroscopy, and Leica MZ10 F (Leica) equipped with AxioCam HRc CCD camera (Carl Zeiss). Fluorescent images for cryostat sections were obtained using an Axioplan 2 microscope with Apotome system (Carl Zeiss), and LSM5 Pascal confocal laser scanning microscope (Carl Zeiss). Images were further processed with LSM Image Browser software (Carl Zeiss) or Axio vision (Carl Zeiss). For confocal microscopy with a thick section, Z-stack spanning 70 μm was reconstructed out of 100 μm.

## Supporting Information

S1 Movie A three-dimensional reconstruction image of fluorescent ink-infused INVP using a thick transverse section of E5 spinal cord. This image was obtained by LSM5 Pascal confocal laser scanning microscope, and processed with LSM Image Browser software. Z-stack for 70 μm.
(MOV)
